# The Value of Alkaline Phosphatase-to-Albumin Ratio in Detecting Synchronous Metastases and Predicting Postoperative Relapses among Patients with Well-Differentiated Pancreatic Neuroendocrine Neoplasms

**DOI:** 10.1155/2020/8927531

**Published:** 2020-02-06

**Authors:** Wentao Zhou, Yuan Fang, Xu Han, Tiantao Kuang, Xuefeng Xu, Wenhui Lou, Dansong Wang

**Affiliations:** ^1^The Research Institution of General Surgery, Zhongshan Hospital, Fudan University, Shanghai 200032, China; ^2^Department of Pancreatic Surgery, Zhongshan Hospital, Fudan University, Shanghai 200032, China

## Abstract

**Backgrounds:**

Pancreatic neuroendocrine neoplasm (pNEN) is a highly heterogeneous entity, presenting widely varied biological behavior as well as long-term prognosis. Reliable biomarkers are urgently needed to make risk stratifications for pNEN patients, which could be beneficial to the development of individualized therapeutic strategy in the clinical practice. Here, we aimed to evaluate the predictive and prognostic roles of serum alkaline phosphatase-to-albumin ratio (APAR) in well-differentiated pNEN patients.

**Methods:**

We retrospectively analyzed the pathologically confirmed grade 1/2 pNEN patients, who were originally treated in our hospital from February 2008 to April 2018. Univariate and multivariate analyses were performed to assess the value of APAR in detecting synchronous metastases and predicting relapses following curative resections.

**Results:**

A total of 170 eligible cases were included into analysis. Logistic univariate analysis indicated APAR (*P*=0.002) was significantly associated with synchronous distant metastasis among well-differentiated pNEN patients, which was further demonstrated to be an independent risk factor by multivariate analysis (odds ratio 8.127, 95% confidence interval (CI) 2.105–31.372, *P*=0.002) was significantly associated with synchronous distant metastasis among well-differentiated pNEN patients, which was further demonstrated to be an independent risk factor by multivariate analysis (odds ratio 8.127, 95% confidence interval (CI) 2.105–31.372, *P*=0.002) was significantly associated with synchronous distant metastasis among well-differentiated pNEN patients, which was further demonstrated to be an independent risk factor by multivariate analysis (odds ratio 8.127, 95% confidence interval (CI) 2.105–31.372, *P*=0.002) was significantly associated with synchronous distant metastasis among well-differentiated pNEN patients, which was further demonstrated to be an independent risk factor by multivariate analysis (odds ratio 8.127, 95% confidence interval (CI) 2.105–31.372, *P*=0.002) was significantly associated with synchronous distant metastasis among well-differentiated pNEN patients, which was further demonstrated to be an independent risk factor by multivariate analysis (odds ratio 8.127, 95% confidence interval (CI) 2.105–31.372,

**Conclusion:**

APAR may work as a convenient pretreatment marker to detect synchronous distant metastasis for well-differentiated pNEN patients and predict recurrences for curatively resected cases without nerve or vascular invasion. However, these findings should be further verified in prospectively well-designed studies.

## 1. Introduction

Pancreatic neuroendocrine neoplasm (pNEN) is the second largest entity of pancreatic tumors, increasing with the advancements in the diagnostic technology as well as the popularization of health examinations [1, 2]. The vast majority of pNENs are well differentiated, whose clinical outcomes are much better compared to poorly differentiated counterparts' [3]. However, due to the high heterogeneity, even the pathologically well-differentiated lesions present varied biological behavior and different prognosis, which could affect the strategy-making of treatment along with follow-up. Thus, reliable biomarkers are needed to reflect and predict the course of development in well-differentiated pNEN patients.

Alkaline phosphatase (ALP) is a common biochemical indictor of liver function tested at routine admission, which could be secreted by normal tissues including liver, bone, small intestine, and kidney and elevated when these tissues were impacted by trauma, inflammation, metabolic disorder, as well as malignancy [4]. A few previous studies have proved that ALP could be a parameter for progression, therapeutic response and prognosis in various digestive system tumors [5–7]. More recently, Jiménez-Fonseca et al. [8] reported that elevated serum ALP was an independent risk factor for decreased long-term survival among patients with well-differentiated metastatic pNENs. Albumin (ALB), another critical liver function index, has been widely recognized as an evaluation tool for nutritional and inflammatory status [9, 10]. A large number of serum ALB-based markers, such as *γ*-glutamyltransferase-to-albumin ratio, fibrinogen-to-albumin ratio, C-reactive protein-to-albumin ratio, have been demonstrated to be promising noninvasive biomarkers for prognosis in many malignancies [11–13]. Recently, several reports have indicated that the ratio of ALP and ALB could be a novel prognostic predictor for urothelial, hepatocellular, as well as pancreatic cancers [14–16]. However, to date, the predictive role of ALP-to-ALB ratio (APAR) has not been explored or reported in pNEN patients.

Thus, in the present study, we aimed to test the value of APAR in predicting synchronous distant metastases in patients with well-differentiated pNENs. Additionally, we also investigated its role in predicting recurrences among the nonmetastatic cases in the above cohort undergoing curative resections.

## 2. Methods

Pathological data about pNEN patients treated in Zhongshan Hospital, Fudan University from February 2008 to April 2018 were carefully reviewed. One hundred and ninety-eight well-differentiated cases diagnosed according to the 2010 WHO criteria were included into further analysis [17]. The exclusion criteria were described as follows: (1) no liver function test performed within 2 weeks before treatment (*n* = 10); (2) previous history of or concurrent any other malignancies (*n* = 9); (3) presenting obstructive jaundice (*n* = 4); (4) infected by hepatitis B virus (*n* = 5). A total of 170 eligible patients were selected into final analysis.

The demographic, clinical, pathological, and laboratory data of selected cases were extracted from the electronic medical record system of our hospital. The TNM stages were defined based on the 8^th^ version of classification system by the American Joint Committee on Cancer [18]. Follow-up work was conducted by reviewing the outpatient information system or performing telephone interviews. Recurrence-free survival (RFS) for curatively resected patients was defined as the period from the operation day to the date of local recurrence or distant metastasis detected by contrast enhanced imaging or last contact. Overall survival (OS) was considered as the interval between the operation day and the date of death or last contact. These cases were followed up until February 1, 2019, or their death.

This research was approved by the Ethics Committee of Zhongshan Hospital, Fudan University, which was conducted according to the norm of the Declaration of Helsinki.

### 2.1. Statistical Analysis

Continuous and categorical variables were described as medians (interquartile ranges [IQR]) and frequencies (percentages), respectively. The optimal cutoff values of continuous variables including age, tumor diameter, ALB, ALP, and APAR for predicting synchronous metastasis and recurrence were determined through the receiver operating characteristic (ROC) curves, which were calculated and plotted by the MedCalc Statistical Software version 18.2.1. (MedCalc Software bvba, Ostend, Belgium; http://www.medcalc.org). Cumulative survival curves were generated by the Kaplan–Meier method, and the differences between two groups were assessed using the log-rank test. Multivariate analyses for synchronous distant metastasis and RFS were conducted by the logistic regression model and cox proportional hazards model with the forward method (likelihood-ratio test) for variables with *P* < 0.05 in the univariate analyses, respectively. These statistical analyses were accomplished by the SPSS software version 25.0 (IBM Corp., Armonk, NY, USA) and a two-side *P* value < 0.05 was considered as statistically significant.

## 3. Results

### 3.1. Patient Characteristics

The whole cohort (*n* = 170) comprised 81 (47.6%) males and 89 (52.4%) females, with a median age of 52-years-old (IQR 43–61 years) at diagnosis. The vast majority of the patients (77.1%) harbored nonfunctioning tumors and more than half of the lesions (60.6%) located at the body and tail of the pancreas. A total of 166 (97.6%) cases received surgical treatments, and 158 (92.9%) and 8 (4.7%) of them underwent curative and palliative resections, respectively. Among the pancreatic lesion resected patients (*n* = 165), 21 (12.7%) were proven to have nerve invasion, 26 (15.8%) were vascular invasion, and 15 (9.1%) were lymph node metastasis, pathologically. According to the WHO grading and AJCC staging criteria, there were 73 (42.9%) G1 and 97 (57.1%) G2 cases, respectively, and 138 (81.2%) stage I-II and 30 (17.6%) stage III-IV cases, respectively. Detailed clinicopathological characteristics were summarized in Table 1.

### 3.2. Risk Factors of Synchronous Distant Metastasis for Well-Differentiated pNEN Patients

In this study, there were 17 (10.0%) patients diagnosed with distant metastasis synchronously. Fifteen of them were only detected with liver metastasis, whereas the rest 2 cases had multiple organs involved. The optimal cutoff points of age, tumor diameter, ALB, ALP, and APAR for predicting synchronous metastasis were 56 years, 3.6 cm, 39 g/L, 90 U/L, and 1.98, respectively, which were calculated by the ROC curves (Supplementary [Supplementary-material supplementary-material-1]). Logistic univariate analysis indicated that age (*P*=0.024), pancreatic lesion location (*P*=0.029), tumor diameter (*P*=0.010), histological grade (*P*=0.011), ALB (*P*=0.019), ALP (*P*=0.004), and APAR (*P*=0.002) were significantly associated with synchronous distant metastasis among well-differentiated pNEN patients. However, only age (odds ratio (OR) 0.045, 95% confidence interval (CI) 0.005–0.416, *P*=0.006), location (OR 11.214, 95% CI 2.061–61.026, *P*=0.005), grade (OR 15.414, 95% CI 1.769–134.320, *P*=0.013), and APAR (OR 8.127, 95% CI 2.105–31.372, *P*=0.002) were demonstrated to be independent risk factors by multivariate analysis (Table 2).

### 3.3. Risk Factors of Recurrence for Nonmetastatic pNEN Cases Undergoing Curatively Surgical Resections

**Figure 1:**
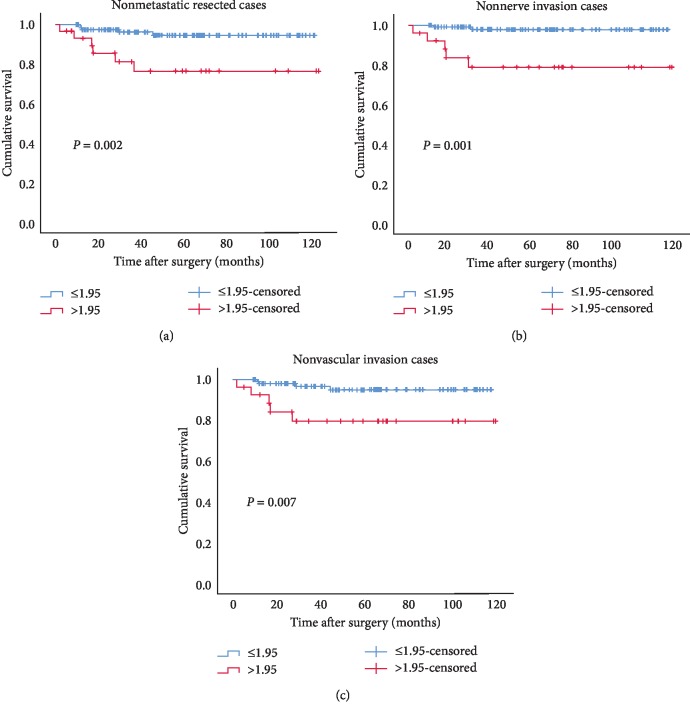
Kaplan–Meier curves for recurrence-free survival stratified by APAR in the nonmetastatic pNEN patients undergoing resections (a) and in the subgroups of pathologically confirmed nonnerve invasion (b) and nonvascular invasion (c) cases. APAR, alkaline phosphatase-to-albumin ratio; pNEN, pancreatic neuroendocrine neoplasm.

We further evaluated the predictive role of APAR for recurrence among nonmetastatic patients following curative resections (*n* = 151). The median follow-up period for these cases was 48.2 months (IQR 26.7–76.7 months). At the last follow-up, 11 patients were detected with recurrences radiologically, 131 had no positive finding, and 9 could not be contacted. The optimal cutoff values of age, tumor diameter, ALB, ALP, and APAR for predicting recurrence were 54 years, 3.6 cm, 41 g/L, 79 U/L, and 1.95, respectively (Supplementary [Supplementary-material supplementary-material-1]). Cox univariate analysis showed that diameter (*P*=0.004), nerve invasion (*P*=0.004), lymph node metastasis (*P* < 0.001), histological grade (*P*=0.022), TNM stage (*P* < 0.001), ALP (*P*=0.008), as well as APAR (*P*=0.007) were statistically associated with RFS for nonmetastatic resected pNEN patients. Survival curves calculated by Kaplan–Meier method also indicated that patients with APAR >1.95 had shorter RFS (*P*=0.002, Figure 1(a)). However, multivariate analysis found that only nerve invasion (hazard ratio (HR) 3.939, 95% CI 1.093–14.198, *P*=0.036) and lymph node metastasis (HR 10.237, 95% CI 2.956–35.456, *P* < 0.001) were independent predictors in this cohort (Table 3). For the OS, no significant difference was found between APAR >1.95 and APAR ≤1.95 groups (*P*=0.898, Supplementary [Supplementary-material supplementary-material-1]).

### 3.4. Subgroup Analysis of the Predicting Role of APAR for RFS

Subgroup analysis was performed to assess the value of APAR in predicting RFS among the above resected cohort. APAR >1.95 was shown to be associated with poor RFS in the subgroups of male (*P*=0.036), age >54 years (*P*=0.018), nonfunction (*P*=0.008), diameter >3.6 cm (*P*=0.032), no cystic component (*P*=0.014), single lesion (*P*=0.023), no necrosis (*P*=0.008), no nerve invasion (*P*=0.004), no vascular invasion (*P*=0.015), as well as G2 grade (*P*=0.012) via univariate analyses, whereas multivariate analyses showed APAR was an independent predictor only in the cases without nerve invasion (HR 7.685, 95% CI 1.433–41.209, *P*=0.017) or vascular invasion (HR 4.789, 95% CI 1.241–18.473, *P*=0.023) (Table 4, Figures 1(b) and 1(c)).

## 4. Discussion

PNENs are highly heterogeneous and even among the morphologically well-differentiated cases, a certain part could progress early and rapidly, which implies the significance of individualized diagnosis and treatment with the guidance of reliable biomarkers. Alkaline phosphatase-to-albumin ratio, as a novel and convenient index derived from routine liver function test, has been discovered to be independently related to prognosis in many malignancies following various anticancer therapies in recent reports [14–16, 19].

In the present study, we found APAR significantly increased in the well-differentiated pNEN patients harboring synchronous distant metastases, which means it could serve as a potential marker to differentiate metastatic and nonmetastatic cases prior to treatment. Moreover, in the prognostic analyses, elevated APAR was shown to be associated with poor RFS in the nonmetastatic cohort receiving curatively surgical treatments, but it was not an independent predictor verified by the multivariate analysis. Additionally, further subgroup analysis indicated APAR could act as an independent prognostic indicator in the cases without nerve invasion or vascular invasion, respectively, implying it still could be a promising biomarker for prognosis among these pathologically specific patients.

ALP is a hydrolase playing the physiologic roles in the dephosphorylation and transphosphorylation processes of many molecules in alkaline conditions. It is mainly synthesized in liver, bone and several other organs, whose serum concentration increases when the corresponding tissues are impaired under a number of pathological circumstances including tumor metastasis. Zhou et al. [20] reported that ALP was an independent risk factor for synchronous bone metastases in lung cancer, and Li et al. [21] found that serum ALP elevated along with the growth of liver metastases in the rat model with colon cancer. Except for the metabolic disorders of specific tissues affected by metastatic lesions, the ALP elevation could be attributed to the tumor's self-secretion, which has been reported in renal and gastric cancers [22]. Thus, ALP could be regarded as a tumor-associated antigen, and accumulating evidences indicated it could promote tumor progression by regulating cell cycle and participating in epithelial-mesenchymal transition (EMT) [23, 24]. A large prospective study conducted by Fairweather et al. [25] showed that metastatic neuroendocrine tumor patients with elevated ALP suffered significantly shorter median OS compared to the counterparts with normal levels (38.4 vs 98.5 months). A similar observation was recently obtained in an international multicenter cohort consisted of well-differentiated metastatic pNENs [8]. As another part of APAR parameter, ALB has already been demonstrated to be a critical risk factor associated with the prognosis in various cancers. However, the interactional mechanism between ALB and tumor is quite complicated and not entirely clear. Generally, tumor can directly inhibit the synthesis of ALB by releasing proinflammatory cytokines or make the ALB permeate into interstitial space with the vascular permeability's increase in systemic inflammation response, causing low serum ALB level [26]. Subsequently, low ALB weakens the body's antitumor reaction and deceases the response to antitumor therapy, contributing to the dismal prognosis of the patients [27]. Recently, Li et al. [28] preformed the analysis on prognosis-related factors among patients with hepatic and pancreatic neuroendocrine tumors, and the results suggested that ALB < 35 g/L was a strong prognostic indicator for long-term survival.

To our knowledge, we analyze the diagnostic and prognostic roles of APAR in the pNEN cohort for the first time. As we mentioned above, APAR could be a useful marker to help detect synchronous metastases in pNEN patients. Thus, an elevated APAR in the pretreatment test could work as an important reminder for more detailed systemic evaluation, such as positron emission tomography-computed tomography (PET-CT) or positron emission tomography-magnetic resonance imaging (PET-MRI). Moreover, careful intraoperative exploration is also essential for such cases undergoing tentatively curative surgeries since metastatic patients can still gain favorable long-term outcomes following radical resections. Liver is the most common metastatic site of pNEN, which needs to be paid great attention to for evaluating as well as exploring. We could not assess the relation between APAR level and liver specific metastases here because almost all (15/17, 88.2%) metastatic cases in our study are hepatic metastases. Larger cohorts containing enough cases with different metastatic tissues should be set up to measure the role of APAR in detecting organ specific metastases among pNEN patients. For prognostic value, though APAR is not independently associated with recurrences in nonmetastatic patients receiving curative resections, we found APAR was still a promising indicator for relapses among patients harboring no pathological nerve or vascular invasion. Thus, intensive follow-up, especially with abdominal imaging, should be considered for early detection and intervention in these subgroups. No statistical association was discovered between APAR and OS, partly due to the relatively good biological behavior of well-differentiated pNENs, resulting in limited endpoint events. Further survival analysis will be performed with longer follow-up interval in the future.

Several limitations should be discussed. As a retrospective, single-center study, selection bias exists. Large-scale multicenter studies with optimal designs are needed to verify the predictive value of APAR. Moreover, the median follow-up time of 48.2 months may not be enough to reflect the entire course of well-differentiated pNEN, considering its favorable prognosis, and further analysis should be performed in the future. Additionally, though inclusion and exclusion criteria have been set up in the present research, the serum levels of ALP and ALB still could be impacted in other physical or pathological conditions.

## 5. Conclusions

APAR could probably work as a convenient pretreatment marker to detect synchronous distant metastasis for well-differentiated pNEN patients, which is easily obtained from routine serological examinations. In addition, it could also play prognostic roles in predicting recurrences for curatively resected patients with no pathologically confirmed nerve or vascular invasion. However, prospectively well-designed studies should be performed to test these findings in the near future.

## Figures and Tables

**Table 1:** Clinicopathological characteristics of patients with well-differentiated pNENs.

Characteristics	
Gender, male, *n* (%)	81 (47.6)
Age, years (median, IQR)	52 (43–61)
Nonfunctioning tumor, *n* (%)	131 (77.1)
Primary tumor location, *n* (%)	
Head-neck	65 (38.2)
Body-tail	103 (60.6)
Both	2 (1.2)
Primary tumor diameter, cm (median, IQR)	2.7 (1.5–4.5)
Single pancreatic lesion, *n* (%)	162 (95.3)
Cystic component, *n* (%)	23 (13.5)
Surgery, *n* (%)	
Curative	158 (92.9)
Palliative	8 (4.7)
No	4 (2.4)
Necrosis, *n* (%)	5 (3.0)^*∗*^
Nerve invasion, *n* (%)	21 (12.7)^*∗*^
Vascular invasion, *n* (%)	26 (15.8)^*∗*^
Lymph node metastasis, *n* (%)	15 (9.1)^*∗*^
Histological grade, *n* (%)	
G1	73 (42.9)
G2	97 (57.1)
AJCC stage, *n* (%)	
I-II	138 (81.2)
III-IV	30 (17.6)
Unknown	2 (1.2)
Synchronous distant metastasis, *n* (%)	17 (10.0)
Albumin, g/L (median, IQR)	41 (39–43)
Alkaline phosphatase, U/L (median, IQR)	64 (56–77)
Alkaline phosphatase-to-albumin ratio (median, IQR)	1.62 (1.31–1.92)

PNEN, pancreatic neuroendocrine neoplasm; AJCC, American Joint Committee on Cancer; IQR, interquartile range. ^*∗*^Evaluated in patients undergoing pancreatic lesion resections (*n* = 165).

**Table 2:** Risk factor analysis for synchronous distant metastasis in pNEN patients.

Characteristics	Univariate analysis		Multivariate analysis	
	OR (95% CI)	*P*	OR (95% CI)	*P*
Gender (male vs. female)	0.974 (0.357–2.658)	0.959		
Age (>56 vs. ≤56 years)	0.094 (0.012–0.729)	0.024	0.045 (0.005–0.416)	0.006
Nonfunctioning (yes vs. no)	1.037 (0.318–3.384)	0.952		
Location (body-tail vs. head-neck)	5.369 (1.186–24.316)	0.029	11.214 (2.061–61.026)	0.005
Diameter (>3.6 vs. ≤3.6 cm)	4.010 (1.401–11.480)	0.010	*—*	NS
No. of primary lesions (multiple vs. single)	1.304 (0.151–11.280)	0.810		
Cystic component (yes vs. no)	0.372 (0.047–2.950)	0.349		
Histological grade (G2 vs. G1)	14.222 (1.840–109.934)	0.011	15.414 (1.769–134.320)	0.013
ALB (>39 vs. ≤39 g/L)	0.292 (0.104–0.814)	0.019	*—*	NS
ALP (>90 vs. ≤90 U/L)	5.416 (1.738–16.873)	0.004	*—*	NS
APAR (>1.98 vs. ≤1.98)	5.022 (1.781–14.164)	0.002	8.127 (2.105–31.372)	0.002

PNEN, pancreatic neuroendocrine neoplasm; ALB, albumin; ALP, alkaline phosphatase; APAR, alkaline phosphatase-to-albumin ratio; OR, odds ratio; CI, confidence interval; NS, not significant.

**Table 3:** Cox analysis of predictors for recurrence-free survival in resected pNEN patients.

Characteristics	Univariate analysis		Multivariate analysis	
	HR (95% CI)	*P*	HR (95% CI)	*P*
Gender (male vs. female)	0.948 (0.289–3.109)	0.930		
Age (>54 vs. ≤54 years)	2.651 (0.776–9.064)	0.120		
Nonfunctioning (yes vs. no)	0.307 (0.039–2.397)	0.260		
Location (body-tail vs. head-neck)	0.819 (0.237–2.834)	0.753		
Diameter (>3.6 vs. ≤3.6 cm)	7.140 (1.893–26.930)	0.004	*—*	NS
Cystic component (yes vs. no)	0.039 (0.000–54.195)	0.379		
No. of primary lesions (multiple vs. single)	2.256 (0.289–17.636)	0.438		
Necrosis (yes vs. no)	*—*	0.705		
Nerve invasion (yes vs. no)	6.058 (1.758–20.875)	0.004	3.939 (1.093–14.198)	0.036
Vascular invasion (yes vs. no)	1.605 (0.346–7.439)	0.546		
Lymph node metastasis (yes vs. no)	13.137 (3.966–43.519)	<0.001	10.237 (2.956–35.456)	<0.001
Histological grade (G2 vs. G1)	11.057 (1.412–86.573)	0.022	*—*	NS
AJCC stage (III vs. I-II)	11.307 (3.431–37.269)	<0.001	*—*	NS
ALB (>41 vs. ≤41 g/L)	0.144 (0.018–1.127)	0.065		
ALP (>79 vs. ≤79 U/L)	5.018 (1.530–16.454)	0.008	*—*	NS
APAR (>1.95 vs. ≤1.95)	5.192 (1.584–17.015)	0.007	*—*	NS

PNEN, pancreatic neuroendocrine neoplasm; AJCC, American Joint Committee on Cancer; ALB, albumin; ALP, alkaline phosphatase; APAR, alkaline phosphatase-to-albumin ratio; HR, hazard ratio; CI, confidence interval; NS, not significant.

**Table 4:** Stratification analysis of APAR for recurrence-free survival in resected pNEN patients.

APAR >1.95 vs. ≤1.95	Univariate analysis		Multivariate analysis	
	HR (95% CI)	*P*	HR (95% CI)	*P*
Gender				
Male (*n* = 71)	6.767 (1.130–40.518)	0.036	—	NS
Female (*n* = 80)	4.013 (0.809–19.902)	0.089		
Age				
≤54 years (*n* = 87)	2.000 (0.208–19.227)	0.548		
>54 years (*n* = 64)	7.283 (1.411–37.587)	0.018	—	NS
Nonfunctioning				
No (*n* = 116)	5.326 (1.541–18.407)	0.008	—	NS
Yes (*n* = 35)	—	0.600		
Location				
Head-neck (*n* = 62)	4.812 (0.804–28.807)	0.085		
Body-tail (*n* = 87)	3.853 (0.643–23.083)	0.140		
Diameter				
≤3.6 cm (*n* = 105)	8.908 (0.807–98.321)	0.074		
>3.6 cm (*n* = 46)	4.614 (1.140–18.670)	0.032	—	NS
Cystic component				
No (*n* = 129)	4.438 (1.354–14.547)	0.014	—	NS
Yes (*n* = 22)	—	—		
No. of primary lesions				
Single (*n* = 144)	4.205 (1.217–14.529)	0.023	—	NS
Multiple (*n* = 7)	—	0.594		
Necrosis				
No (*n* = 147)	5.009 (1.528–16.415)	0.008	—	NS
Yes (*n* = 4)	—	—		
Nerve invasion				
No (*n* = 134)	11.466 (2.224–59.130)	0.004	7.685 (1.433–41.209)	0.017
Yes (*n* = 17)	1.003 (0.103–9.804)	0.998		
Vascular invasion				
No (*n* = 131)	5.136 (1.379–19.131)	0.015	4.789 (1.241–18.473)	0.023
(*n* = 20)	5.831 (0.347–97.939)	0.221		
Lymph node metastasis				
No (*n* = 139)	2.286 (0.418–12.493)	0.340		
Yes (*n* = 12)	6.078 (0.674–54.824)	0.108		
Histological grade				
G1 (*n* = 71)	—	0.758		
G2 (*n* = 80)	5.112 (1.442–18.124)	0.012	—	NS
AJCC stage				
I (*n* = 52)	—	0.756		
II (*n* = 86)	3.466 (0.579–20.750)	0.173		
III (*n* = 13)	7.332 (0.813–66.154)	0.076		

PNEN, pancreatic neuroendocrine neoplasm; AJCC, American Joint Committee on Cancer; ALB, albumin; ALP, alkaline phosphatase; APAR, alkaline phosphatase-to-albumin ratio; HR, hazard ratio; CI, confidence interval; NS, not significant.

## Data Availability

The data used to support the findings of this study are available from the corresponding author upon request.

## Supplementary Materials

Supplementary Materials The optimal cutoff values of albumin, alkaline phosphatase, alkaline phosphatase-to-albumin ratio, age and tumor diameter in detecting synchronous metastasis among patients with well-differentiated pNENs (A) and in predicting recurrence among resected pNEN patients (B) determined by the receiver operating characteristic curves. pNEN, pancreatic neuroendocrine neoplasm. Supplementary Figure 2. Kaplan–Meier curves for overall survival stratified by APAR in the nonmetastatic pNEN patients undergoing resections. APAR, alkaline phosphatase-to-albumin ratio; pNEN, pancreatic neuroendocrine neoplasm
