# Supplementary figures and images for: The Value of Alkaline Phosphatase-to-Albumin Ratio in Detecting Synchronous Metastases and Predicting Postoperative Relapses among Patients with Well-Differentiated Pancreatic Neuroendocrine Neoplasms
Source: J Oncol. 2020 Feb 6;2020:8927531. doi: 10.1155/2020/8927531 (PMC7026734; doi:10.1155/2020/8927531)

## Supplementary Figure 1

**A**

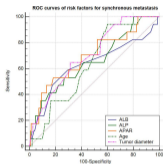

**B**

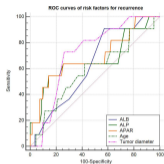

## Supplementary Figure 2

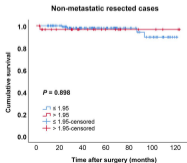

Supplement: Supplementary Materials — The optimal cutoff values of albumin, alkaline phosphatase, alkaline phosphatase-to-albumin ratio, age and tumor diameter in detecting synchronous metastasis among patients with well-differentiated pNENs (A) and in predicting recurrence among resected pNEN patients (B) determined by the receiver operating characteristic curves. pNEN, pancreatic neuroendocrine neoplasm. Supplementary Figure 2. Kaplan–Meier curves for overall survival stratified by APAR in the nonmetastatic pNEN patients undergoing resections. APAR, alkaline phosphatase-to-albumin ratio; pNEN, pancreatic neuroendocrine neoplasm [file 8927531.f1.pdf]
